# The role of early intergroup experiences for identification with all humanity in adulthood

**DOI:** 10.3389/fpsyg.2023.1042602

**Published:** 2023-03-15

**Authors:** Katarzyna Hamer, Sam McFarland

**Affiliations:** ^1^Institute of Psychology of Polish Academy of Sciences, Warsaw, Poland; ^2^Western Kentucky University, Bowling Green, KY, United States

**Keywords:** identification with all humanity, global human identification, childhood, adolescence, early intergroup experiences, intergroup contact, parental styles

## Abstract

Identification with all humanity (IWAH), defined as a bond with and concern for people all over the world, predicts concern for global problems, commitment to human rights, and prosocial activities. However, it is still unknown how such a broad social identification develops and if early experiences play any role. Two studies explored the role of diverse childhood and adolescence intergroup experiences in predicting IWAH in adulthood. We focused on experiences such as being raised in diversity and having intergroup friends, helping or being helped by various others, and having experiences leading to re- or de-categorization, and introduced a new Childhood/Adolescent Intergroup Experiences (CAIE) scale. Study 1 (*N* = 313 U.S. students, *M_*age*_* = 21) and Study 2 (*N* = 1,000, a representative Polish sample, *M_*age*_* = 47) found that this kind of intergroup experiences during childhood and adolescence predicted IWAH beyond the effects of its other known predictors, such as empathy, openness to experience, universalism, right-wing authoritarianism, social dominance orientation or ethnocentrism. These results, obtained on various samples and in countries with different ethno-cultural contexts, point to potential ways of enlarging IWAH during childhood and adolescence.

## 1. Introduction

Global social identifications [such as identification with all humanity (IWAH) or global citizenship] are concepts of the growing interest of researchers, practitioners, and the general population (for a review, see McFarland et al., 2019), and in recent decades there has been a surge in calls for education for “global citizenship” (UNESCO, 2014, n.d.). The relevance of studying and developing them worldwide is of growing importance, especially in our world fighting global problems which can be solved only by a global effort, such as climate change, refugee crisis, hunger, or the spread of the COVID-19 pandemic. And still, nationalism and a backlash against globalization have been increasing in many places (see, e.g., Huddy et al., 2021; Pástor and Veronesi, 2021). In this context, global human identification, Gandhi’s belief that “All humanity is one undivided and indivisible family” can be seen as an important contrasting ideal.

The concept of identifying with all humanity has been present in psychological theories for decades. All humanity is considered the highest level of possible self-categorization in self-categorization theory (Turner, 1987). It also appeared in the classical works of Maslow (1954), where it was considered the highest level of self-actualization; Adler (1927/1954), where it was a characteristic of the most mature individuals; Allport (1958) who claimed that without humanity becoming a common ingroup human conflict is endless and inevitable, and Erikson (1968) who perceived it as the desired direction of identity development. McFarland et al. (2012; 2019) describe IWAH as reflecting an active caring and concern for people all over the world and regarding them as members of one’s ingroup. Assessed by the IWAH scale, it is a relatively stable individual disposition, distinct from personality, empathy, or universalism, which are among its predictors (McFarland et al., 2012, 2019; Hamer et al., 2019).

Global social identifications can be studied as individual dispositions or made situationally salient (see more in Hamer et al., 2019). In this manuscript, we use the former approach, aiming to explore the potential role of childhood and adolescence intergroup experiences in developing dispositional IWAH. More specifically, we ask if this broad identification can be enlarged by such experiences during childhood or adolescence to become a quality of individuals as adults. Because IWAH embodies a sense of connection with all people, regarding them as members of one’s own group and within one’s range of concern, early social intergroup experiences can likely either enhance or hinder its development. So far, despite researchers’ efforts, there are hardly any results showing significant connections between early experiences and IWAH (for a review, see McFarland et al., 2019). Our paper brings a new contribution to this topic. In two cross-sectional studies (on 313 U.S. students and a representative sample of 1,000 Polish citizens) conducted in countries with different ethno-cultural contexts (see, e.g., Hamer et al., 2020), we test early experiences that might predict IWAH in adulthood, exploring socialization, as well as broader social experiences from school, neighborhood, and community, including interactions with adults, peers, and observations of interactions of others. Thus, we explore not only parental childrearing styles, which so far were not proven to be significant IWAH’s predictors (McFarland et al., 2019) or weakly connected at best (Hamer, 2017) but also diverse intergroup experiences from childhood and adolescence that might have helped individuals become more aware of the common humanity and open up to diverse others and their needs, no matter what their “race” or ethnic and cultural background. Since such a broad social identification most probably emerges later in social (see, e.g., Maslow, 1954; Allport, 1958; Erikson, 1968) and cognitive development (Corenblum and Armstrong, 2012), we conducted our studies on adults, asking about their early experiences and measuring their IWAH.

### 1.1. Identification with all humanity

Individuals who strongly identify with all humanity declare feeling close to people all over the world, caring for them, and perceiving them as a single ingroup. IWAH predicts support for universal human rights, concern for global problems such as the spread of potentially dangerous diseases around the planet (e.g., COVID pandemic, Zika, etc.), refugee cris.es, poverty, global warming, and willingness to work toward solving them, an array of prosocial activities from donating blood and volunteering to humanitarian helping and supporting international charities (Hamer and Baran, 2022, July; McFarland, 2017a; McFarland et al., 2019; Sparkman and Hamer, 2020), support for refugees (Bassett and Cleveland, 2019), cooperative health behavior and willingness to vaccinate during the COVID-19 pandemic (Barragan et al., 2021; Deng, 2021; Murphy et al., 2021; Marchlewska et al., 2022; Sparkman, 2022), sustainable behaviors (see, e.g., Der-Karabetian et al., 2014, 2018; Loy et al., 2021, 2022; Pong, 2021), and forgiveness toward former national enemies (Hamer et al., 2017, 2018). IWAH is also connected to greater knowledge of global humanitarian issues and actively learning about them, less tendency to dehumanize outgroups, and lower Islamophobia (Dunwoody and McFarland, 2018; McFarland et al., 2019; Sparkman and Hamer, 2020). A laboratory study showed the positive role of IWAH while performing tasks in inter-ethnic dyads (Hamer et al., 2022). Thus, enlarging this broad identification can be seen as potentially beneficial for societies and humanity (see e.g., UNESCO, 2014).

IWAH is also connected to political attitudes and collective action. While it gives mixed results regarding the connection to political orientations in different countries, studies conducted by Hamer and McFarland showed its negative relation with support for building a wall on the U.S.-Mexican border and isolationism and positive relation to granting U.S. citizenship to “Dreamers” and internationalism regarding humanitarian aid (McFarland et al., 2019). In the context of the COVID pandemic, Lantos et al. (2022) demonstrated that IWAH led not only to donation intentions toward vulnerable groups but also predicted political action intention to put pressure on politicians to make the best decisions for the community and challenge bad decisions.

Earlier research has shown that IWAH is positively predicted by dispositional empathy, openness to experience, and universalism and negatively predicted by ethnocentrism and its two main roots (Altemeyer, 1998), right-wing authoritarianism (RWA), and social dominance orientation (SDO; Hamer et al., 2019). It is negatively correlated with blind patriotism, indices of self-centeredness, psychopathy, and Machiavellianism (McFarland et al., 2012, 2019). It is related also to the positively perceived impact of globalization around the world, on one’s own country, and world-minded value orientation (i.e., a concern for the welfare of people around the world, sharing of resources, tolerance of diversity, and international cooperation; see Der-Karabetian et al., 2018).

But how can global human identification be developed? Do early intergroup experiences contribute to IWAH in adults? So far, only Hamer (2017) found a weak but significant correlation between IWAH and the father and mother giving autonomy and acceptance to a child for both student and representative national Polish samples. McFarland et al. (2013) found no relationships between seven general parenting factors (e.g., punitive, affectionate, spoiling, religious) and IWAH. We decided to explore this issue further, thus focusing on diverse types of early intergroup experiences rather than just general parenting styles, exploring both socialization and broader social experiences from school, neighborhood, and community.

### 1.2. Potential role of early social experiences

There are empirical and theoretical reasons to think that early social experiences can shape openness toward other people and, thus, the breadth of social identifications. Evidence suggests that children show signs of intergroup biases from early childhood and that these biases increase with age (Raabe and Beelmann, 2011; Skinner and Meltzoff, 2019). Ingroup favoritism among children appears early: 2 days-old newborns already prefer to listen to the speech stream of their native language (Moon et al., 1993), while 3-month-olds prefer own-race faces (Bar-Haim et al., 2006). Further, 4-year-olds show biases in favor of own-gender children (e.g., Cvencek et al., 2011), and 5-year-olds prefer to befriend native speakers of their own language than children with foreign accents (Kinzler et al., 2009). In studies by Renno and Shutts (2015), “White” 3-year-olds gave more resources to unfamiliar “White” than to unfamiliar “Black” children and to unfamiliar same-gender than unfamiliar other-gender children. Chinese and African pupils ages 4–5 attending ethnically homogeneous or heterogeneous kindergartens already correctly identified themselves with their own ethnic group and recognized the ethnicity of dolls (De Caroli et al., 2013). McLoughlin et al. (2018) noted childhood dehumanization of members of geographically-based or gender outgroups: 5- and 6-year-olds presented explicit ingroup preferences, while 6-year-olds also showed a tendency to perceive ambiguous morph faces as less human when they belonged to an outgroup. In another study, 6-year-olds produced a greater diversity of mental state terms when talking about their own social group (McLoughlin and Over, 2017). An international study showed that preference for the national ingroup appears by 6 years of age in all national groups (Barrett et al., 2004; see also Ruble et al., 2004). Therefore, it can be concluded that research demonstrated ingroup favoritism appearing already during childhood.

Moreover, developmental psychologists argue that this early stage of life is an optimal developmental phase to intervene upon intergroup biases before these become entrenched patterns of thought and behavior, as it is harder to change these biases in adulthood (Lee et al., 2017; Skinner and Meltzoff, 2019). A few studies proved it to be successful. Bar-Haim et al. (2006) experimentally showed that preference for own-race faces is moderated by infants’ exposure to members of other races in the immediate social environment – when it is diverse, there is no ingroup preference. Other research has shown the effectiveness of extended contact (knowing of ingroup members being friends with outgroup members) even for 5-year-olds, which improved their attitudes toward refugees (Cameron et al., 2006). Based on this research, it is plausible that early experiences that expose children and young adolescents to positive contact with persons of different groups can help offset these intergroup biases and provide a foundation for IWAH.

### 1.3. What type of experiences during childhood and adolescence can help build identification with all humanity?

Using an example of ethnic identity, Corenblum and Armstrong (2012) argue that in children and young adolescents, the social identity reflects attitudes and emotional reactions of significant others, their socialization efforts, as well as broader acculturation processes present at school, in neighborhoods, and community. We believe it may also be true for IWAH. Below we present the results of our search for potential clues on what type of experiences during childhood and adolescence can help build IWAH in three areas: (a) research on intergroup contact, (b) research on the influence of adults regarding intergroup attitudes (thus understood broader than just parental styles), (c) early experiences described in biographies of people meritorious for human rights or saving others’ lives.

#### 1.3.1. Intergroup contact

Intergroup contact has long been considered one of the most effective strategies for improving intergroup relations (Dovidio et al., 2003; Pettigrew and Tropp, 2006). The basic assumption of the intergroup contact theory is that “interactions between members of different groups improve attitudes toward the other group(s) and thus reduce intergroup tensions. The contact approach provides a clear and concise guideline for systematic interventions: to improve interethnic relations, persons with different ethnic backgrounds should be brought in direct (i.e., face-to-face) contact or should experience indirect (i.e., contact without face-to-face interactions)” (Lemmer and Wagner, 2015, p. 152) or imaginary contact (see a meta-analysis by Miles and Crisp, 2014; see also Smith and Minescu, 2022).

As the meta-analysis by Pettigrew et al. (2011) shows, intergroup contact typically reduces prejudice through increased empathy and reduced anxiety in intergroup interactions. Among the positive outcomes, there is also increased intergroup trust. Moreover, these effects typically generalize to the whole outgroup, other situations, and even to other outgroups not involved in the contact. These positive outcomes also appear to be universal and replicated across nations, genders, and age groups. Since more prejudiced people are less prone to include all humans in their group (see, e.g., Sparkman and Hamer, 2020), it is reasonable to assume that early intergroup contact, which lowers prejudice and increases trust, may also have positive effects on forming IWAH.

The quality of contact appears important as well: Positive experiences and cooperation with outgroup members reduce intergroup bias more than do less positive experiences (Pettigrew et al., 2011; Skinner and Meltzoff, 2019). Studies by Wolsko et al. (2003) and Voci and Hewstone (2003) reveal how intergroup contact not only reduces prejudice but also undermines group stereotypes, increasing the perceived variability of persons in outgroups. However, a review of the effects of intergroup contact on ethnic relations prepared by Amir (1969) showed that only “favorable” conditions tend to reduce prejudice, while “unfavorable” tend to increase intergroup tension and prejudice.

Additionally, interpersonal interactions enable de-categorization, leading to greater attention to the unique attributes of team members instead of focusing on their outgroup membership (Ensari and Miller, 2001). Studies show the benefits of teaching de-categorization (for primary school pupils, see, e.g., Jones and Foley, 2003). Interpersonal interactions also enable seeing shared commonalities, thus recategorization, which means adopting a superordinate level of categorization, common for both ingroup and outgroup. Common Ingroup Identity Model (Gaertner et al., 1989; Gaertner and Dovidio, 2000) research shows the beneficial effects of strengthening one-group representations while weakening separate-group representations. It demonstrates how the situational activation of a broad social identification (e.g., with “humans”) positively affects attitudes toward former outgroup members, who now become fellow members of a common ingroup. Eller and Abrams (2003) found that more intergroup contact in social settings predicted weaker separate group representations, which in turn predicted lower levels of prejudice. Stronger superordinate, one-group representations predicted lower levels of intergroup anxiety and more favorable outgroup evaluations. Levin et al. (2003) longitudinal study revealed that a common ingroup identity (i.e., a one-group representation) leads, over time, to an increased formation of “outgroup” friendships.

As various meta-analyses have shown, intergroup friendship is particularly important in reducing prejudice (e.g., Pettigrew et al., 2011), especially when cross-group friendships involve socializing and more time spent together (Davies et al., 2011). Even having a friend who has an outgroup friend (thus, indirect contact) reduces prejudice (Pettigrew et al., 2011).

Based on these considerations, we hypothesize that early intergroup contact would contribute to developing IWAH.

There is also research directly linking intergroup contact to IWAH, although in adulthood. German participants who imagined engaging in a simulated chat with a Paraguayan had higher IWAH scores as compared to a control group (Römpke et al., 2018). Studies on U.S. samples found that having more contact and experiences with people from foreign cultures and their cultural elements (art, music, cuisine, etc.) was associated with a stronger bond with all humanity (Sparkman and Eidelman, 2018), and studies on a Polish nationwide sample confirmed these results also for concern for all humanity (Sparkman and Hamer, 2020). A study on IWAH and traveling (Loy et al., 2021) showed that the quantity and experienced quality of contact with local people met during international journeys were positively related to IWAH. Further, increasing international salience *via* exposure to posters of the globe or flags of different countries increased concern with all humanity compared to a control group and resulted in giving more money to global and local charities (Reese et al., 2015). Similar results were obtained for other measures of global attachment: increasing international salience *via* exposition to news about People’s World Peace Project (an organization working for a global peace community) caused a significant increase in the sense of global community compared to ordinary news or news about fashion (de Rivera and Mahoney, 2018).

Since we know from these studies that intergroup contact in adulthood (real or imagined) is positively connected to IWAH (or even has the potential to enlarge it), it is time to research its potential role in childhood and adolescence, which is the aim of our current research.

#### 1.3.2. The influence of adults

Children often acquire biased or unbiased attitudes and behaviors from observing the behavior of trusted adults, from parental socialization messages and unintended cues (e.g., non-verbal signals; Skinner and Meltzoff, 2019). Research has shown that children’s attitudes toward others are more affected by parents’ behaviors than by their words. Parental intergroup friendships are associated with less intergroup bias among children (Pahlke et al., 2012), while there is only a weak relationship between the memories of parents’ providing children with information about intergroup relations and children’s prejudices (Skinner and Meltzoff, 2019). On the other hand, studies have found that children’s implicit (but not explicit) racial biases were consistent with their parent’s explicit racial biases (Sinclair et al., 2005; Pirchio et al., 2018). Other results have shown that children’s explicit intergroup biases were consistent with parents and other close adults’ implicit intergroup biases (Skinner and Meltzoff, 2019).

Teaching values and empathy (especially if not only by words but also modeled) can also be beneficial (Stepien and Baernstein, 2006; UNESCO, 2014; Malti et al., 2016). According to Duriez et al. (2005, p. 317), “educational programs that try to tackle the societal problem of prejudice by promoting certain values will never be entirely successful unless they focus simultaneously on the promotion of Openness to Change and Self-Transcendence values.” Brito-Pons et al. (2018) found that adults’ participation in a 9 week Compassion Cultivation Training program that taught empathy, embracing shared common humanity and compassion toward all beings, enhanced both participants’ empathic concern and IWAH scores.

On the basis of these considerations, we assume that parents/caretakers having intergroup friendships, promoting being open to all, intended or unintended, and teaching empathy and universalistic values can contribute to developing IWAH in children and adolescents. We would distinguish, however, between parents’ general styles of childrearing and their specific teaching of empathy and openness toward all. From previous research, we know that RWA and SDO are IWAH’s predictors (Dunwoody and McFarland, 2018; Hamer et al., 2019; McFarland et al., 2019). On the other hand, Duckitt (2001) claimed that punitive socialization lies in the roots of RWA, while unaffectionate socialization lies in the roots of SDO. Thus, we use Duckitt’s socialization practices scale to test if parental practices (punitive and unaffectionate) or maybe rather early experiences significantly contribute to IWAH in adulthood. We hypothesize that whether parents are strict or lenient, affectionate or unaffectionate, may be less important for the development of IWAH (see McFarland et al., 2019) than the specific opportunities for intergroup contact and teachings they impart.

#### 1.3.3. Experiences of people meritorious for human rights or saving others

While reading biographies or interviews with individuals who were meritorious for human rights or helping others during WW2, one often sees sentences showing their high IWAH. Monroe (1996) summarizes her interviews with altruists who rescued Jews and others during WW2 as follows:

*Altruists seem to conceive of themselves as part of all mankind rather than as members of any particular group or subgroup. This perception of themselves as part of common humanity and not personalistic or empathic ties to family, gender, and religious, national, or ethnic groups, most aptly captures the systematic and consistent differences between paradigmatic rational actors and altruists (Monroe, 1996, p. 204).*

*The idea of being welded together, of belonging to one human family, surfaced over and over again in my interviews; indeed, I was struck by the similarity of expressions used* (Monroe, 1996, p. 205).

Individuals distinguished for their contributions to human rights also show their connection with all humanity in writings, interviews, or memoirs (McFarland, 2022). What patterns can one discover in their early experiences, potentially enlarging their social IWAH? Below we present our conclusions from analyzing selected examples of biographies of (1) people meritorious for human rights (chosen from the book *Heroes of Human Rights* by McFarland, 2022) or (2) people known for risking their lives to save “others.”

*Being raised or educated in diversity* and *having intergroup friends* are common memories of people who saved the lives of others during WW2 or/and were meritorious for contributions to human rights. We find these recollections in the lives of Raphael Lemkin, who coined the term “genocide” and fought for many years to make it an international crime (McFarland and Hamer, 2016), Japanese consul Chiune Sugihara (later known as Sempo Sugiwara), who saved around 10,000 Jews by giving them Japanese visas during WW2 to escape to safety (Levine, 2019), Irena Sendler, Polish Righteous Among the Nations, who was a mastermind behind the action to rescue several hundreds of Jewish children from the Warsaw ghetto during WW2 (Mieszkowska, 2018), and Jean Henry Dunant, who led in creating the First Geneva Convention, on treating war wounded, requiring that both the enemy’s and one’s own wounded to be treated, and the International Red Cross to provide this treatment (Moorehead, 1999).

Another recurring memory is *being moved by seeing the suffering* of other people (including outgroups), which relates to what we can label as empathy-related responding (Malti et al., 2016). Raphael Lemkin remembered being deeply moved by the suffering of others while as a child reading about the atrocities of the Romans toward the Christians under Nero, or “the French king, who watched the hanging of the Huguenots from his balcony. Ordered more light on the scene so that he might better see the tormented faces of the dying.” (McFarland and Hamer, 2016, p. 70). While still a child, Lemkin heard of a pogrom against Jews in Bialystok, a nearby city, in which “the mobs opened the stomachs of their victims and stuffed them with the feathers from the pillows and the feather comforters.” (McFarland and Hamer, 2016, p. 70). From these experiences, Lemkin learned that “a line of blood led from the Roman arena through the gallows of France to the pogrom at Bialystok.” (McFarland and Hamer, 2016, p. 70). As a result, he “grew up with a strong sense of feeling that persecution must cease and that justice and love will finally prevail” (Lemkin, 2013, pp. 17–18). This belief was strengthened while learning about the massacre of more than 1,200,000 Armenians committed by the Turkish Interior Minister Pasha, which led Lemkin to coin the term “genocide” and to lead the struggle to create the 1948 U.N. *Convention on the Prevention and Punishment of the Crime of Genocide* (McFarland and Hamer, 2016).

Being moved by seeing the suffering of others appears also in Henry Dunant’s memories of the horrors of the 1859 Battle of Solferino (McFarland, 2017b) and of Chiune Sugihara, who, during his service in Manchuria, could not stand Japanese atrocities toward Chinese and, as an act of protest, submitted his resignation (Gmitrzak, 2010). The later suffering of Jews fleeing Poland during WW2 was Sugihara’s main reason for granting them Japanese visas against the decision of his own government, which ruined his diplomatic carrier and risked his life (Levine, 2019). As he said, “After struggling and agonizing, I concluded that humanity is paramount. Then, fearing nothing, I decided to issue those visas” (Sugihara, n.d.).

*Adults teaching children and adolescents to pay attention to the suffering of others and to be empathetic toward all* is another common pattern we find in these biographies. Sugihara attended the famous Goko national academy founded by Shimpei Goko, who also established its code to take care of others and not expect rewards for one’s own goodness (Levine, 2019). Helping others in need was an important value during Henry Dunant’s childhood in Geneva. He had seen his parents help the sick, poor, orphans, and prison parolees. Following their example, as a late teen, he joined the Geneva Society for Alms Giving, volunteering time to care for the sick and poor and prisoners.

Eleanor Roosevelt, one of the key authors of the *Universal Declaration of Human Rights*, also as a child learned about the suffering of those around her and was taught to help:

*Very early I became conscious of the fact that there were people around me who suffered in one way or another. I was five or six when my father took me to help serve Thanksgiving dinner in one of the newsboys’ clubhouses. My father explained that many of these ragged little boys had no homes and lived in little wooden shanties in empty lots, or slept in vestibules of houses or public buildings, anyplace where they could be moderately warm. I was not in ignorance that there were sharp contrasts, even though our lives were blessed with plenty* (Roosevelt, 1961, pp. 12−13).

As a result, Roosevelt devoted great effort throughout her life to ending the suffering and mistreatment of others, both for fellow Americans and for all people (McFarland, 2014).

We find a similar pattern in other biographies. When Robert Bernstein, a founder of Human Rights Watch was just 10 years old, a governess who worked for his wealthy New York City family took him on weekends to Central Park where they would visit encampments of “men and women wearing ragged clothes stooped over little cooking fires.” As he wrote in his autobiography, “I began to realize that a lot of people had it very rough in the world” (Bernstein, 2016, p. 5). Bernstein carried this lesson with him throughout his life, leading him to create Human Rights Watch, which monitors human rights abuses around the world.

Irena Sendler, known for saving more than 2,500 children from the Warsaw Ghetto during WW2, remembered that her parents’ house was always open for those in need. Her physician father often treated poor farmers and Jews free of charge, saying that if someone needed help, one needed to provide it. She recalled: “I was raised to believe that the question of religion, nation, belonging to any race is of no importance – it’s a human being that matters!” (Dziȩciołowska, 2018). As a child, she played with poor Jewish friends. Later, at Warsaw University, she opposed the practice of segregating Jewish students. During WW2, she helped both wounded Polish soldiers and Jews, who were then officially served only by the Jewish institutions. Later, she helped smuggle clothing, food, and other necessities into the Warsaw Ghetto. Risking her life, she helped the Jews who escaped the ghetto and helped smuggle children out of it.

Not only intergroup contact but also *being helped by an outgroup member* appears to be another factor enabling common ingroup identification of humans. An example may be the experience of Franz Uri Boas, later known as the “Father of American Anthropology,” whose crusade against “scientific” racism was a breakthrough in modern anthropology. Being 23 years old at the time, on his first expedition to study the Inuit Eskimo on Baffin Island, he once wandered lost and frozen. The Inuit saved him, taking him into a warm igloo and feeding him, sharing what they had. This experience made Boas see the humanity of the peoples he would continue to study. He wrote, “the Eskimo is a man as we are; his feelings, his virtues, and his shortcomings are based on human nature, like ours” (Hyatt, 1990, p. 10).

These abovementioned early life experiences are biographical examples of how social identity could be shaped by socialization and broader social experiences in families, at schools, in neighborhoods, and community. We decided to include these types of experiences in our studies and check if they can be connected to IWAH in adulthood.

### 1.4. Current research

The goal of the current research was to empirically test the connection between recollections of childhood and adolescent intergroup experiences that might have the effect of increasing awareness of common humanity and concern for a wider range of people, fostering the development of dispositional IWAH as a quality seen in adulthood. We decided to study adults and assess their memories because, as we have mentioned before, such a broad social identification most probably emerges later in social (see, e.g., Maslow, 1954; Allport, 1958; Erikson, 1968) and cognitive development (Corenblum and Armstrong, 2012).

We included a range of intergroup experiences, such as interactions with adults and peers and observations of interactions of others. Based on the considerations presented in the introduction (see section 1.3), we created Childhood/Adolescent Intergroup Experiences (CAIE) scale depicting experiences that could have the possible effect of increasing awareness of common humanity and concern for a wider range of people, enabling the development of IWAH. We chose those which were found as important in the research we described in the introduction, that repeatedly appeared in numerous biographies, or were considered almost as forming experiences (such as being saved by an outgroup member).

More specifically, we included items regarding experiences related to being raised in diversity and having intergroup friends (“*I grew up having friends from different cultural or/and ethnic background from my own*,” *“I grew up in an environment with people from different cultural or/and ethnic background present or visiting”*), being taught empathy (“*My parents/caregivers taught me how to be empathetic toward all people*”) and compassion for all, also by experiencing suffering of others (“*I deeply experienced suffering of someone from a different cultural or/and ethnic background during my childhood or adolescence;*” “*During my childhood or adolescence I did not like it when a person from different cultural or/and ethnic background was discriminated against.*”), experiences involving helping or being helped by various others (“*During my childhood or adolescence, a person from a different cultural or ethnic background helped me when I needed support*,” *“During my childhood or adolescence a person from different cultural or/and ethnic background helped a person from my group when s/he needed support*”), and those leading to re- or de-categorization (“*During my childhood or adolescence, I had an experience showing me that skin color, attractiveness, body built, or other ‘trivial’ differences between people do not really matter compared to their personality*,” “*I had an “opening up” experience with people from different cultural or/and ethnic background during my childhood or adolescence”*).

In the following studies, we tested if such experiences predict IWAH beyond the possible role of general parenting styles (study 1) and known IWAH predictors (study 1 and 2), such as empathy (especially perspective taking and empathic concern), openness to experience, the value of universalism, right-wing authoritarianism (RWA), social dominance orientation (SDO), and ethnocentrism (see Hamer et al., 2019; McFarland et al., 2019).

## 2. Study 1

We formed three hypotheses to test in this study:

H1. Adults with more childhood and adolescent intergroup experiences opening them up to others will have higher IWAH than adults with fewer experiences of this kind.
H2. These kinds of childhood and adolescent intergroup experiences contribute more to IWAH in adulthood than do general parenting styles.
H3. These kinds of childhood and adolescent intergroup experiences contribute to IWAH in adulthood beyond other predictors of IWAH, including openness to experience, dispositional empathy, universal values, right-wing authoritarianism, social dominance orientation, and ethnocentrism.

### 2.1. Method

#### 2.1.1. Participants

The participants consisted of 313 U.S. junior and senior students at a public university in Kentucky, 72% female, ages 18–73 (*M* = 21, *SD* = 5.5), 88% White, 3.5% African-American, 2.6% Hispanic, 2.2% Asian, 2.2% mixed, all U.S. citizens, who took part in an anonymous survey *via* Survey Monkey. Students could participate in a drawing for cash prizes as a reward for taking part in this study.

#### 2.1.2. Measures

##### 2.1.2.1. Early intergroup experiences

To measure childhood and adolescent intergroup experiences, we used our own nine-item Childhood/Adolescent Intergroup Experiences (CAIE) scale, described in section 1.4 (for the whole scale, see the Supplementary material 1.1). The scale assesses the adults’ memories of their own intergroup experiences from childhood and adolescence that might have the effect of increasing awareness of common humanity and concern for a wider range of people. The responses were coded on a 4-point scale from 1 (*This statement does not describe me at all*) to 4 (*This statement very much describes me*).

To examine the structure of the CAIE scale in the U.S. sample, we subjected all items to a principal components analysis (PCA) using direct oblimin rotation (see Table 1, and for full details see Supplementary material 3). Results of a scree plot suggest a multi-component solution (the eigenvalue of the first component was 3.56, with 40% of variance explained). Although no clear second factor was found, we decided to remove the items which loaded on the first factor < 0.4. It left us with six items out of the original 9. This time factor analysis showed a clear single-component solution (see Table 2) with an eigenvalue of 3.21, with 53.5% of variance explained. The mean scores of the six items constituted the CAIE-USA composite score. Cronbach’s α for the shortened 6-item scale was = 0.82.

**TABLE 1:** Structure matrix for the full 9-item CAIE scale (study 1, the USA, and study 2, Poland).

	USA			Poland	
Structure matrix	Component			Component	
	**1**	**2**	**3**	**1**	**2**
1. I grew up having friends from different cultural or/and ethnic background from my own.	**0.797**	0.107	0.4	**0.821**	0.305
2. My parents/caregivers taught me to be empathetic toward all people.	0.122	0.789	−0.059	0.343	0.885
3. I had an “opening up” experience with people from different cultural or/and ethnic background during my childhood or adolescence.	**0.421**	0.241	0.767	**0.814**	0.463
4. I deeply experienced suffering of someone from different cultural or/and ethnic background during my childhood or adolescence.	0.307	−0.035	0.816	**0.791**	0.476
5. I grew up in an environment with people from different cultural or/and ethnic background present or visiting.	**0.82**	0.151	0.311	**0.87**	0.397
6. During my childhood or adolescence a person from different cultural or/and ethnic background helped me when I needed support.	**0.858**	0.192	0.312	**0.873**	0.356
7. During my childhood or adolescence I did not like it when a person from different cultural or/and ethnic background was discriminated against.	**0.502**	0.567	0.086	**0.691**	0.57
8. During my childhood or adolescence a person from different cultural or/and ethnic background helped a person from my group when s/he needed support.	**0.808**	0.233	0.256	**0.868**	0.345
9. During my childhood or adolescence I had an experience showing me that skin color, attractiveness, body built or other “trivial” differences between people do not really matter compared to their personality.	0.215	0.693	0.445	**0.519**	0.828

Extraction method: Principal component analysis. Rotation method: Oblimin with Kaiser Normalization. Loadings > 0.4 in the first factor are bolded.

**TABLE 2:** Component matrix for the shortened CAIE-USA (study 1) and CAIE-PL (study 2) scales.

Component matrix^a^		
	Component	Component
	USA	Poland
I grew up having friends from different cultural or/and ethnic background from my own.	0.807	0.799
I grew up in an environment with people from different cultural or/and ethnic background present or visiting.	0.804	0.861
During my childhood or adolescence a person from different cultural or/and ethnic background helped me when I needed support.	0.834	0.854
During my childhood or adolescence a person from different cultural or/and ethnic background helped a person from my group when s/he needed support.	0.786	0.849
I had an “opening up” experience with people from different cultural or/and ethnic background during my childhood or adolescence.	0.570	0.818
During my childhood or adolescence I did not like it when a person from different cultural or/and ethnic background was discriminated against.	0.524	0.727
I deeply experienced suffering of someone from different cultural or/and ethnic background during my childhood or adolescence.	–	0.806
During my childhood or adolescence I had an experience showing me that skin color, attractiveness, body built or other “trivial” differences between people do not really matter compared to their personality.	–	0.614

Extraction method: principal component analysis. ^a^, 1 components extracted.

##### 2.1.2.2. Identification with all humanity (IWAH)

IWAH was assessed with McFarland et al.’s (2019) original nine-item IWAH scale [e.g., “*How close do you feel to each of the following groups:*” (a) People in my community, (b) Americans, (c) people all over the world (scale: 1 - *not at all* to 5 - *very close*); “*How often do you use the word ‘we’ to refer to the following groups of people?*’ (a) People in my community, (b) Americans, and (c) people all over the world (scale: 1 - *almost never* to 5 - *very often*)]. Mean scores from identification with “people all over the world” subscale constitute “raw” scores of IWAH (α = 0.81). However, following Dunwoody and McFarland (2018), we additionally used the “pure” IWAH measure to control for the IWAH correlations with remaining identifications (the mean of the IWAH items was regressed onto the means of the other identifications, and the standardized residual was the “pure” IWAH measure). We additionally analyzed the results of IWAH scale looking at its two subscales: bond with (items 1–4; α = 0.71), and concern for (items 6−9, α = 0.78) all humanity (see more in: Hamer et al., 2021).

##### 2.1.2.3. Openness to experience

To measure openness to experience, we used 10 openness-to-experience items from a 60-item version of the HEXACO Personality Inventory-Revised (Ashton and Lee, 2009; e.g., “*I’m interested in learning about the history and politics of other countries*,” “*I like people who have unconventional views*”). The responses were coded on a 5-point scale from 1 (*strongly disagree*) to 5 (*strongly agree*). Cronbach’s α = 0.80.

##### 2.1.2.4. Universalism

To measure universalism, we used nine items from the universalism subscale of Schwartz’s Portrait Values Questionnaire (Cieciuch and Schwartz, 2012; e.g., “*It is important to him/her to be tolerant toward all kinds of people and groups”*). Responses to the PVQ were made on a 6-point Likert-like scale ranging from 1 (*not like me at all*) to 6 (*very much like me*). Cronbach’s α = 0.83.

##### 2.1.2.5. Empathy

To assess dispositional empathy, we used the two highly correlated subscales from the Interpersonal Reactivity Index (IRI; Davis, 1994), previously found to predict IWAH (see Hamer et al., 2019): (1) perspective taking, defined as a tendency to spontaneously consider the psychological viewpoints of others (e.g., “*When I’m upset at someone, I usually try to ‘put myself in his shoes’ for a while*”), and (2) empathic concern, defined as a tendency to experience feelings of sympathy or compassion for unfortunate others (e.g., “*I often have tender, concerned feelings for people less fortunate than me*”). Each subscale consisted of seven items with a 5-point response scale ranging from 1 (*does not describe me well*) to 5 (*describes me well*). Cronbach’s α for the 14-item scale = 0.83.

##### 2.1.2.6. Right-wing authoritarianism

Right-wing authoritarianism was measured using a 12-item version of Funke’s (2005) three-dimensional RWA scale. It assesses three components of authoritarianism: aggression (e.g., “*What our country really needs is a strong, determined leader which will crush the evil and set us on our right way again*”), submission (e.g., “*Obedience and respect for authority are the most important values children should learn*”) and conventionalism (e.g., “*The withdrawal from tradition will turn out to be a fatal fault one day*”). The responses were coded on a 5-point scale from 1 (*strongly disagree*) to 5 (*strongly agree*). Cronbach’s α for the full scale was = 0.87.

##### 2.1.2.7. Social dominance orientation

Social dominance orientation was assessed using a 15-item SDO scale (Pratto et al., 1994; e.g., “*It’s ok if some groups have more of a chance in life than others*”; Cronbach’s α = 0.92). The responses were coded on a 5-point scale from 1 (*strongly disagree*) to 5 (*strongly agree*).

##### 2.1.2.8. Ethnocentrism

To assess the ethnocentrism of participants, defined as unfavorable attitudes toward other groups, we used a short 6-item version of the Manitoba Ethnocentrism Scale (Altemeyer, 1996; e.g., “*It is simply a waste of time to train some races for good jobs; they simply don’t have the drive and determination it takes to learn a complicated skill*”; Cronbach’s α = 0.77). The responses were coded on a 5-point scale from 1 (*strongly disagree*) to 5 (*strongly agree*).

##### 2.1.2.9. Parenting socialization styles

We assessed the students’ memories of their parents or other caregivers’ socialization styles using Duckitt‘s (2001) measures of punitive and unaffectionate parenting styles. The six-item punitive style measure (Cronbach’s α = 0.62) consists of three items measuring harsh parenting (e.g., “*I was often physically punished in a painful manner while growing up*”) and three assessing strict parenting (e.g., “*I was strictly disciplined while growing up*”), although the two subscales load on a single factor. The items from Duckitt’s seven-item measure of unaffectionate socialization (e.g., “*I grew up in an unaffectionate environment*”) load on a second factor (Cronbach’s α = 0.86). The responses were coded on a 4-point scale from 1 (*This statement does not describe me at all*) to 4 (*This statement very much describes me*), same as CAIE.

### 2.2. Results

Table 3 provides the correlations, means, and standard deviations of the measured variables. An inspection of the correlations reveals significant negative correlations between early intergroup experiences measured by CAIE-USA and RWA, and ethnocentrism (and statistical trend for SDO), and positive correlations between early intergroup experiences and all remaining variables: IWAH (both “pure” and “raw” scores), openness to experience, empathy (perspective taking and empathic concern) and universalism. All earlier predictors of IWAH (both “raw” and “pure” scores) correlated with it again in this sample. IWAH or CAIE-USA did not correlate with punitive and unaffectionate parenting socialization styles.

**TABLE 3:** Correlations, means, and standard deviations for measured variables (study 1).

Variables	1	2	3	4	5	6	7	8	9	10	11
1. IWAH “pure” scores	-	0.90 **	0.26 **	0.31**	-0.41**	-0.32**	-0.41**	0.46**	0.001	0.07	0.37**
2. IWAH “raw” scores		-	0.23**	0.39**	-0.30**	-0.13*	-0.31**	0.44**	-0.04	-0.02	0.25**
3. CAIE-USA			-	0.19**	-0.20**	-0.11*	-0.09^t	0.25**	0.07	0.06	0.17**
4. Empathy				-	-0.29**	-0.05	-0.38**	0.46**	-0.06	-0.05	0.29**
5. Ethnocentrism					-	0.53**	0.65**	-0.48**	0.07^t	-0.03	-0.38**
6. RWA						-	0.45**	-0.48**	0.03	-0.18**	-0.45**
7. SDO							-	-0.55**	0.05	-0.04	-0.36**
8. Universalism								-	-0.04	0.07	0.44**
9. Punitive P									-	0.51**	0.12*
10. Unaffected P										-	0.18**
11. Openness											-
*M*	0.11	3.42	2.62	3.82	2.85	2.83	1.92	4.54	2.14	1.64	3.52
*SD*	0.99	0.60	0.65	0.50	0.67	0.77	0.69	0.75	0.66	0.60	0.66

N = 313, M, mean; SD, standard deviation; ** p < 0.01,* p < 0.05, ^t^p < 0.1 (1-tailed). IWAH, identification with all humanity; CAIE, Childhood/Adolescent Intergroup Experiences scale; RWA, right wing authoritarianism; SDO, social dominance orientation; Punitive P, punitive parental style; Unaffected P, unaffected parental style.

We conducted hierarchical regression analysis upon IWAH^1^, in the first step considering all predictors apart from early experiences: openness, dispositional empathy, universalism, authoritarianism, social dominance, and ethnocentrism. In the second step, we added all variables measuring early experiences (parental styles and CAIE-USA) to see how they would contribute to the explained variance. As shown in Tables 4a, b, in regression analyses using either “pure” or “raw” IWAH scores, early experiences as assessed by the CAIE-USA predicted higher IWAH, confirming Hypothesis 1. These early intergroup experiences measure still positively predicted IWAH (both “raw” and “pure” scores) beyond the effects of openness, dispositional empathy, universalism, authoritarianism, social dominance, and ethnocentrism, confirming Hypothesis 3.

**TABLE 4a:** Predictors of identification with all humanity (IWAH; “raw scores,” Study 1).

	Model 1			Model 2		
Variables	*β*	*B (SE)*	95% CI	*β*	*B (SE)*	95% CI
Intercept		1.27 (0.41)	[0.46, 2.09]		1.22 (0.42)	[0.38, 2.05]
Openness to experience	0.06	0.06 (0.05)	[−0.05, 0.16]	0.06	0.06 (0.05)	[−0.05, 0.16]
Empathy	0.18**	0.21 (0.07)	[0.07, 0.35]	0.16**	0.20 (0.07)	[0.06, 0.34]
Universalism	0.33***	0.26 (0.05)	[0.15, 0.37]	0.31***	0.23 (0.06)	[0.14, 0.35]
RWA	0.15*	0.11 (0.05)	[0.01, 0.22]	0.14*	0.11 (0.05)	[0.01, 0.21]
SDO	−0.03	−0.02 (0.06)	[−0.14, 0.10]	−0.04	−0.04 (0.06)	[−0.16, 0.08]
Ethnocentrism	−0.13^t^	−0.11 (0.06)	[−0.24, 0.01]	−0.10	−0.09 (0.06)	[−0.22, 0.03]
CAIE-USA				0.10*	0.09 (0.05)	[0.00, 0.19]
Punitive P				−0.02	−0.02 (0.05)	[−0.13, 0.08]
Unaffected P				−0.02	−0.02 (0.06)	[−0.13, 0.10]
Adjusted *R*^2^	0.24			0.25		
*F*	*F* (6,306) = 17.63***			*F* (9,303) = 12.26***		
Δ*R*^2^	0.26			0.01		
Δ*F*				*F* (3, 303) = 1.39		

N = 313, *** p < 0.001,** p < 0.01,* p < 0.05, ^t^p < 0.1. IWAH, identification with all humanity; CAIE, Childhood/Adolescent Intergroup Experiences scale; RWA, right wing authoritarianism; SDO, social dominance orientation; Punitive P, punitive parental style; Unaffected P, unaffected parental style.

**TABLE 4b:** Predictors of identification with all humanity (IWAH; “pure scores,” study 1).

	Model 1			Model 2		
Variables	β	*B (SE)*	95% CI	β	*B (SE)*	95% CI
Intercept		−1.70 (0.67)	[−3.03, −0.38]		−1.97 (0.69)	[−3.33, −0.62]
Openness to experience	0.15**	0.23 (0.09)	[0.06, 0.39]	0.14*	0.21 (0.09)	[0.04, 0.38]
Empathy	0.08	0.17 (0.12)	[−0.06, 0.40]	0.07	0.14 (0.12)	[−0.08, 0.37]
Universalism	0.22**	0.29 (0.09)	[0.12, 0.47]	0.20**	0.26 (0.09)	[0.08, 0.43]
RWA	−0.02	−0.03 (0.08)	[−0.19, 0.14]	−0.02	−0.03 (0.09)	[−0.20, 0.14]
SDO	−0.09	−0.14 (0.10)	[−0.33, 0.06]	−0.12^t^	−0.17 (0.10)	[−0.37, 0.03]
Ethnocentrism	−0.14*	−0.21 (0.10)	[−0.41, −0.01]	−0.12^t^	−0.18 (0.10)	[−0.38, 0.02]
CAIE-USA				0.13**	0.20 (0.08)	[0.05, 0.35]
Harsh P				−0.007	−0.01 (0.09)	[−0.18, 0.16]
Unaffected P				0.02	0.03 (0.10)	[−0.16, 0.22]
Adjusted *R*^2^	0.27			0.28		
*F*	*F* (6,306) = 20.58***			*F* (9,303) = 14.69***		
Δ*R*^2^	0.29			0.02		
Δ*F*				*F* (3, 303) = 2.36^t^		

N = 313, *** p < 0.001,** p < 0.01,* p < 0.05, ^t^p < 0.1. IWAH, identification with all humanity; CAIE, Childhood/Adolescent Intergroup Experiences scale; RWA, right wing authoritarianism; SDO, social dominance orientation; Punitive P, punitive parental style; Unaffected P, unaffected parental style.

Early experiences measured by the CAIE-USA correlated with IWAH and predicted it in a regression analysis, while parental socialization styles did not; therefore, Hypothesis 2 was also confirmed.

### 2.3. Discussion

Study 1 confirmed all three hypotheses. The analyses showed a positive relationship between early intergroup experiences (as described by the CAIE-USA scale) and IWAH. However, as expected, no relationship between IWAH and the parenting styles proposed by Duckitt (2001) was found, indicating that general parenting styles, even if underlying the development of RWA and SDO, do not catch the crucial early experiences responsible for developing IWAH. It was also found that early intergroup experiences described by the CAIE-USA positively predicted IWAH beyond the predictive roles of empathy, openness to experience, the value of universalism, RWA, SDO, and ethnocentrism. This is the first study to show a relationship between early social experiences and IWAH (see McFarland et al., 2019). These results indicate that while general parenting styles – harsh or not, strict or lenient, or affectionate or unaffectionate – have little if any effect upon later IWAH, opportunities to personally interact with people of different socio-cultural backgrounds and specific experiences it brings, do enhance thinking of all humanity as part of one’s own ingroup, as members of common humanity.

## 3. Study 2

Study 2 was designed to test: (1) if the CAIE is also a significant predictor of IWAH on a nationwide representative sample of adult participants and (2) in a different socio-cultural and ethnic context. While Study 1 was conducted on a U.S. university student sample, Study 2 was conducted on a representative nationwide sample of 1,000 Polish adult citizens. The United States and Poland differ on many cultural dimensions. One, especially relevant here, is social diversity. The U.S. is rather diverse while Poland is one of the most nationally and religiously homogeneous countries and its index of ethnic cohesion (more than 97%) is one of the highest in the world (Hamer et al., 2020). Will Hypotheses 1 and 3 from Study 1 also be confirmed even in such a homogenous country? We again assume that adults with more childhood and adolescent intergroup experiences have higher IWAH than adults with fewer experiences of this kind, even in such a homogenous country (Hypothesis 1); and that intergroup experiences still contribute to enlarging IWAH beyond other predictors of IWAH, such as openness to experience, dispositional empathy, universal values, RWA, SDO, and ethnocentrism (Hypothesis 3). Because parental socialization styles did not contribute to IWAH in Study 1, were not found predictive of IWAH in earlier studies (see McFarland et al., 2019), and for reasons of length limitations in the nationwide survey, we did not measure them in Study 2, and therefore did not reexamine Hypothesis 2.

### 3.1. Method

#### 3.1.1. Participants

The measures were administered as a part of a larger study^2^ to a nationally representative sample of 1,000 adult Polish citizens from all regions of the country with the Computer Assisted Personal Interviews (CAPI) method. The participants ranged in age from 18 to 84 years (*M* = 47.4, *SD* = 16), with 52% women, all White. The opinion poll company offered them chocolate as a “thank you” for completing the survey.

#### 3.1.2. Measures

We used Polish language adaptations of the same measures as used in Study 1. With the exception of the CAIE, the response scales for all other measures were identical to the U.S. versions.

##### 3.1.2.1. Early intergroup experiences

To measure childhood and adolescent experiences, we used our nine-item Childhood/Adolescent Intergroup Experiences (CAIE) scale in Polish adaptation by the first author (α = 0.91, see Supplementary material 1.2). Responses to the CAIE were made on a 5-point scale ranging from 1 (*does not describe me well*) to 5 (*describes me well*).

To examine the structure of the CAIE scale in the Polish sample, we subjected all items to a principal components analysis (PCA) using direct oblimin rotation, as in Study 1. Results of a scree plot again suggest a single-component solution, which explains a majority of the variance (the eigenvalue of this component was 5.27, 58.5% of variance explained; see Table 1 and Supplementary material 3 for details). Although no clear second factor was found, one item (*My parents/caregivers taught me to be empathetic toward all people*) loaded on the first factor < 0.4 and was removed, following the same strategy as in Study 1. It left us with eight items out of the original nine (see Table 2). The factor analysis for the 8-item scale showed a clear single-component solution with an eigenvalue of 5.06, with 63.16% of variance explained. The mean scores of the remaining 8 items constituted the CAIE-PL composite score. Cronbach’s α for the shortened scale was = 0.91.

##### 3.1.2.2. Universalism

Universalism was measured in the same way as in Study 1, using the Polish adaptation by Cieciuch and Schwartz (2012). Cronbach’s α for the full scale = 0.86.

##### 3.1.2.3. Empathy

Empathy (perspective-taking and empathic concern) was measured in the same way as in Study 1, by two subscales from IRI (Davis, 1994; the Polish adaptation by Kaźmierczak et al. (2007)). Cronbach’s α for the full scale was = 0.74.

##### 3.1.2.4. Identification with all humanity (IWAH)

To measure IWAH, we used the same scale as in Study 1 (IWAH, McFarland et al., 2012) but in the Polish adaptation by the first author (Hamer et al., 2021). Cronbach’s α for the general “raw” IWAH = 0.92, for the IWAH bond = 0.89, and for the IWAH concern = 0.87.

##### 3.1.2.5. Openness to experience

Openness to experience was measured in the same way as in Study 1, using the HEXACO PI-R in Polish adaptation by Szarota (1995; α = 0.71).

##### 3.1.2.6. Right-wing authoritarianism

Right-wing authoritarianism was measured with the shortened scale (Funke, 2005) from Study 1, using six items in Polish adaptation by Bilewicz et al. (2017; α = 0.78).

##### 3.1.2.7. Social dominance orientation

Social dominance orientation was measured using the same 15-item SDO scale (Pratto et al., 1994), using the Polish adaptation by Duriez et al. (2005), modified by the first author to match U.S. items, used in Study 1. Cronbach’s α = 0.65.

##### 3.1.2.8. Ethnocentrism

To assess the ethnocentrism, we used a shortened 3-item Polish version of a Manitoba Ethnocentrism Scale (Altemeyer, 1996) from study 1, in the Polish adaptation by the first author (α = 0.78).

### 3.2. Results

Table 5 provides the correlations, means, and standard deviations of all variables. An inspection of the correlations reveals significant negative correlations between early experiences measured by CAIE-PL with SDO, ethnocentrism (and statistical trend for RWA), and positive correlations between early experiences and IWAH (both “pure” and “raw” scores), openness to experience, empathy (perspective taking and empathic concern), and universalism. Most earlier predictors of IWAH were again significantly correlated with it.

**TABLE 5:** Correlations, means, and standard deviations for measured variables (study 2).

Variables	1	2	3	4	5	6	7	8	9
1. IWAH “pure” scores	-	0.84**	0.19**	0.06*	-0.24**	-0.19**	-0.04	0.11**	0.24**
2. IWAH “raw” scores		-	0.20**	0.24**	-0.16**	-0.04	-0.10**	0.26**	0.29**
3. CAIE-PL			-	0.16**	-0.06*	-0.05^t^	-0.07**	0.10**	0.21**
4. Empathy				-	-0.01	-0.10**	-0.32**	0.55**	0.35**
5. Ethnocentrism					-	0.29**	0.14**	-0.02	-0.17**
6. RWA						-	0.05^t^	-0.15**	-0.18**
7. SDO							-	-0.37**	-0.10**
8. Universalism								-	0.33**
9. Openness to experience									-
*M*	< 0.001	2.91	2.60	3.45	3.33	3.39	2.67	4.48	3.07
*SD*	1.0	0.80	1.0	0.49	0.90	0.72	0.37	0.72	0.59

N = 1,000, M, mean; SD, standard deviation. ** p < 0.01,* p < 0.05, ^t^p < 0.1 (1-tailed). IWAH, identification with all humanity; CAIE, Childhood/Adolescent Intergroup Experiences; RWA, right wing authoritarianism; SDO, social dominance orientation.

As in Study 1, we conducted hierarchical regression analysis upon IWAH^3^, in the first step considering all predictors apart from early experiences: openness, dispositional empathy, universalism, authoritarianism, social dominance, and ethnocentrism. In the second step, we added the measure of the early experiences to see if it would contribute to the explained variance. Tables 6a, b show that early experiences, as assessed by the CAIE-PL, predicted higher IWAH in adulthood for both the “pure” or “raw” IWAH scores and did so beyond the effects of openness, universalism, authoritarianism, social dominance, and ethnocentrism. These results reconfirm Hypotheses 1 and 3, replicating Study 1 results in a different cultural context and on a representative sample of adult citizens.

**TABLE 6a:** Predictors of identification with all humanity (IWAH, “raw scores;” study 2).

	Model 1			Model 2		
Variables	β	*B (SE)*	95% CI	β	*B (SE)*	95% CI
Intercept		1.05 (0.33)	[0.41, 1.69]		0.94 (0.32)	[0.30, 1.57]
Openness to experience	0.19***	0.25 (0.05)	[0.16, 0.34]	0.16***	0.22 (0.05)	[0.13, 0.31]
Empathy	0.09*	0.15 (0.06)	[0.03, 0.27]	0.08*	0.13 (0.06)	[0.01, 0.24]
Universalism	0.16***	0.18 (0.04)	[0.10, 0.26]	0.17***	0.19 (0.04)	[0.10, 0.27]
RWA	0.003	0.003 (0.04)	[−0.07, 0.07]	−0.01	−0.01 (0.04)	[−0.08, 0.06]
SDO	0.03	0.06 (0.07)	[−0.08, 0.20]	0.03	0.07 (0.07)	[−0.07, 0.21]
Ethnocentrism	−0.14***	−0.12 (0.03)	[−0.18, −0.07]	−0.13***	−0.12 (0.03)	[−0.17, −0.06]
CAIE-PL				0.13***	0.11 (0.02)	[0.06, 0.15]
Adjusted *R*^2^	0.13			0.15		
*F*	*F* (6, 993) = 26.49***			*F* (7, 992) = 25.98***		
Δ*R*^2^	0.14			0.02		
Δ*F*				*F* (1, 993) = 19.91***		

N = 1,000, *** p < 0.001,* p < 0.05. IWAH, identification with all humanity; CAIE, Childhood/Adolescent Intergroup Experiences; RWA, right wing authoritarianism; SDO, social dominance orientation.

**TABLE 6b:** Predictors of identification with all humanity (IWAH, “pure scores;” study 2).

	Model 1			Model 2		
Variables	β	*B (SE)*	95% CI	β	*B (SE)*	95% CI
Intercept		−0.37 (0.41)	[−1.18, 0.44]		−0.53 (0.41)	[−1.34, 0.28]
Openness to experience	0.17***	0.29 (0.06)	[0.17, 0.40]	0.14***	0.26 (0.06)	[0.12, 0.35]
Empathy	−0.04	−0.08 (0.08)	[−0.23, 0.07]	−0.06	−0.11 (0.08)	[−0.26, 0.03]
Universalism	0.12**	0.17 (0.05)	[0.06, 0.27]	0.12**	0.17 (0.05)	[0.07, 0.28]
RWA	−0.13***	−0.17 (0.05)	[−0.26, −0.09]	−0.14***	−0.19 (0.04)	[−0.28, −0.11]
SDO	0.04	0.11 (0.09)	[−0.06, 0.29]	0.05	0.13 (0.09)	[−0.05, 0.30]
Ethnocentrism	−0.18***	−0.20 (0.04)	[−0.27, −0.13]	−0.17***	−0.19 (0.04)	[−0.26, −0.13]
CAIE-PL				0.15***	0.15 (0.03)	[0.09, 0.21]
Adjusted *R*^2^	0.11			0.13		
*F*	*F* (6, 993) = 21.88***			*F* (7, 992) = 22.80***		
Δ*R*^2^	0.12			0.02		
Δ*F*				*F* (1, 992) = 25.14***		

N = 1,000, *** p < 0.001, ** p < 0.01. IWAH, identification with all humanity; CAIE, Childhood/Adolescent Intergroup Experiences; RWA, right wing authoritarianism; SDO, social dominance orientation.

### 3.3. Discussion

The results of Study 2 reconfirm two key results: Early intergroup experiences, as assessed by the CAIE-PL, are significantly related to IWAH in adulthood. Second, these intergroup experiences contribute to predicting IWAH beyond the power of its major predictors found in earlier studies. Also, because these results were found in a different culture, they offer evidence of the validity and cross-national consistency of these findings.

## 4. General discussion

Growing global problems, such as climate change, the COVID-19 pandemic, refugee crises, world hunger, and others, urge researchers, international organizations (such as, e.g., UNESCO), and the broader population to look for ways to resolve them. Earlier research shows that collective identity leads to positive actions, helps coordinate and create collective sources of support, mitigates against damaging behaviors, and collectivization in a time of emergency increases the chances of survival (Drury et al., 2019, 2020). In this context, IWAH, one of the broadest social identities, can be recognized as an important ideal to be developed. It predicts support for universal human rights, concern for global problems, positive attitudes toward different groups, and international and national prosocial activities, even in the times of crises such as the COVID-19 pandemic. Individuals who identify with all humanity feel closeness and care toward people all over the world and psychologically include them in their ingroup. The question if such a broad identification can be developed by childhood and adolescent experiences has remained unanswered until now. Despite researchers’ efforts exploring parental styles in this context, almost no connections have been found, apart from only a weak correlation between IWAH and both parents giving autonomy and acceptance to a child and a secure attachment style (Hamer, 2017; see also, McFarland et al., 2013, 2019). Lately, another study was published, showing weak connections between very general “positive parenting behavior” remembered by adult participants and their IWAH (Hagel et al., 2022).

Therefore, we proposed a different approach, expanding our focus from general parental styles to diverse childhood/adolescent intergroup experiences that might increase awareness of common humanity and concern for all people, no matter what their “race” or ethnic and cultural background. We tested early experiences that might predict IWAH in adulthood, exploring socialization, as well as broader social experiences from school, neighborhood, and community, including own interactions and observations of interactions of others that might have had this effect.

We focused on early experiences because research in developmental psychology has shown that childhood is an optimal phase for intervening in intergroup biases before they become entrenched in thought and behavior (Lee et al., 2017; Skinner and Meltzoff, 2019). Intergroup contact is one of the ways to intervene. Experiments on infants show that social diversity can be beneficial from a very early age: seeing the faces of people from different ethnic origins prevents infants’ perceptual narrowing in other-race recognition (Bar-Haim et al., 2006; Anzures et al., 2012). Research on intergroup contact, described in detail in the introduction section, is also very clear on its benefits for lowering stereotypes, prejudice, and intergroup anxiety and for improving intergroup attitudes, trust, and empathy. A few earlier studies showed the possible role of international contact experienced by adults in enlarging their IWAH (see section “1.3.1. Intergroup contact”). Our research has shown the potential benefits of early childhood and adolescence experiences for IWAH.

To find potential early experiences that might enlarge IWAH, we also examined research on parental influence, teaching values, and empathy. Moreover, we analyzed biographies of people meritorious for their human rights contributions or saving others’ lives, who declared feeling closeness to all humanity, to see if we could find similar patterns in their early memories, potentially leading to developing such a broad human identity. On the basis of all these considerations, we introduced a new Childhood/Adolescent Intergroup Experiences (CAIE) scale. The CAIE assesses memory of being raised in diversity, helping or being helped by various others, and having experiences leading to re- or de-categorization. In two studies on different samples and in countries with different ethno-cultural contexts we tested the potential role of such childhood/adolescent experiences in opening up individuals toward others and considering all humanity as part of one’s ingroup.

The results of Study 1, using 313 U.S. university student participants, showed that childhood/adolescent intergroup experiences, as described by the CAIE-USA scale, significantly predicted IWAH and did so beyond the power of the earlier known IWAH predictors, such as empathy, openness to experience, the value of universalism, right-wing authoritarianism (RWA), social dominance orientation (SDO), and ethnocentrism. To our knowledge, this is the first time such results on the connection between early experiences and IWAH have been obtained. In contrast, general parenting styles (punitive or unaffectionate parenting lying in the roots of RWA and SDO; see Duckitt, 2001), which we tested in Study 1, did not predict later IWAH scores (nor any of its factors). In light of previous failed efforts to find strong connections between parental rearing styles and IWAH (for a review, see McFarland et al., 2019), our results once again indicate that general parenting styles do not catch the crucial early experiences that contribute to the development of IWAH. Focusing on parents’ or caregivers’ attitudes and behaviors specifically related to IWAH seems more promising, which our results revealed. Also, Hagel et al. (2022), in a study published lately, showed that the extent to which participants perceived their parents as global citizens explained about one-third of the variance in their own identification as global citizens (for the role of normative environment for developing global citizen identity see also Reysen and Katzarska-Miller, 2013, 2018). Although global human identification and global citizenship identification have some differences in their prototypical meaning (see Carmona et al., 2020), our research, as well as studies by Hagel and colleagues and Reysen and Katzarska-Miller showed that it is not general parenting styles but attitudes and behaviors more specific to global identifications that may be crucial to developing broad social identifications by children and adolescents.

Study 2, conducted on a representative sample of 1,000 adult Polish citizens, reconfirmed key findings from Study 1. Again, childhood/adolescent intergroup experiences, as described by the CAIE-PL, significantly predicted IWAH (and both of its factors) beyond the roles of all other predictors. Our findings from two countries with very different socio-cultural and ethnic contexts (see, e.g., Hamer et al., 2020) reveal an important new predictor of IWAH. Our research also points to a potential way of enlarging IWAH during childhood and adolescence by expanding early intergroup contacts such as being raised in diversity and having intergroup friends, helping or being helped by various others, and having experiences leading to re- or de-categorization. This research is the first in which relationships between early intergroup experiences and later IWAH were found.

The CAIE scale has been demonstrated to be a reliable measure, with no more than one clear factor in both countries. Initial factor analyses on a full 9-item scale showed similar structure and pattern matrixes for both countries, although with some “noise” in the factor structure. Thus, we have decided to remove items loading < 0.4 on the first factor, which resulted in a 6-item scale in the USA and an 8-item-scale in Poland. As a result, we obtained a better, pure one-factor solution in both countries. Interestingly, the deleted item in both countries regarded parents teaching empathy toward all people. Exploratory analyses showed that this item, although positively correlated with IWAH, does not have a predictive role beyond the role of CAIE and other IWAH’s predictors (e.g., see the Supplementary material 5). Thus, our research showed that parental teaching, even if it regards empathy, is less important while developing IWAH than having early intergroup experiences.

In the USA, two other items were removed as loading < 0.4 on the first factor, while they loaded > 0.4 in Poland and were kept. They referred to experiencing the suffering of someone from a different cultural or/and ethnic background during childhood or adolescence and experiencing that personality matters more than skin color, attractiveness, body build, or other “trivial” differences between people. We believe that these differences in the structure of the CAIE scale may be connected to the fact that the USA and Poland have significant differences in the ethno-cultural social setup of their citizens: Poland is ethnically almost homogenous, while the USA is much more diverse. Thus, it is much less common to have certain kinds of direct inter-ethnic experiences in homogenous Poland than in the diverse USA, so when they happen, they may have a bigger influence due to their rarity. Moreover, CAIE predicts general IWAH in both countries, but if we look at the IWAH subscales, it predicts only a bond with all humanity in the USA and both bond and concern for all humanity in Poland. Another reason for these dissimilarities may be connected to the differences in the studied samples: a nationwide representative sample of adult Poles versus a much smaller student sample from one of the universities in Kentucky. Thus, the results from Poland may be more representative of the population. In our opinion, the fact that CAIE clearly predicts general IWAH (and bond with all humanity) in both countries despite their differences in social diversity may indicate its universality; however, more research in other countries and on bigger samples is needed.

Our research has limitations, however difficult to overcome. As we mentioned at the beginning, we formulated hypotheses about early experiences, but our participants were adults, and their experiences were of a retrospective and self-reported nature. Of course, it would be more conclusive to explore children’s experiences as children. However, for developmental reasons, it is not possible in the case of such a broad identification, as with all humanity, because children do not yet have a chance to develop it fully. There is no empirical research concerning how early IWAH can appear; however, according to Erikson’s Theory of Psychosocial Development (1968), it can be expected to occur in middle adulthood. For Maslow (1954), it was the highest level of self-actualization, and for Adler (1927/1954) - a characteristic of the most mature individuals. Research on IWAH shows that students, who are usually young adults, can already exhibit this identification (e.g., McFarland et al., 2012, 2019; Hamer et al., 2017, 2018, 2019), although its level is indeed lower than for older people (see, e.g., Hamer, 2021). Another issue may be a cognitive development process. Since Corenblum and Armstrong (2012) showed its role in the process of developing ethnic identity, we suspect it is similar for developing broader social identifications such as IWAH. Therefore, children most likely cannot be tested for their level of IWAH because developing it is not yet plausible. Future longitudinal studies directly testing intergroup experiences of children and adolescents, followed by measuring the same individuals’ level of IWAH after years, may help overcome these difficulties.

It is also possible that adults with stronger IWAH simply better remember experiences with those of other ethno-cultural backgrounds than do people with weaker IWAH. However, research on intergroup contact and all its positive results give reasons to believe that childhood/adolescent intergroup experiences not only bring less prejudice but, as two sides of the same coin, also inclusion and openness to others so that they help enhance IWAH.

Further, the CAIE scale measures the adults’ memories of their own early experiences, not their parents’ memories. We decided to use this method because previous research has shown a weak relationship between the memories of parents about the information on intergroup relations provided to children and children’s prejudices (see, e.g., Skinner and Meltzoff, 2019). Also, the CAIE scale contains a broader range of experiences, including ones with peers and other adults, at schools, in neighborhoods, and in communities. Many of these experiences may be known only to the individuals themselves.

Nevertheless, if we believe that IWAH is a desirable feature in adults, it is important to study intergroup experiences in childhood and adolescence that might foster its development. We believe that we have shown, albeit by inference from studies with adults, that diverse early intergroup experiences, as contrasted to experiences that are more limited to one’s narrower ingroup, are an important early precursor of later IWAH.

The results obtained in the USA and Poland were consistent in showing that early intergroup experiences measured by CAIE were a significant predictor of IWAH. Moreover, there are positive correlations between CAIE with IWAH, openness to experience, empathy, and universalism, and negative correlations between CAIE with SDO, RWA, and ethnocentrism. However, future studies should further explore the psychometric properties of the CAIE scale and its relations with psychological characteristics, particularly on bigger (ideally nationwide) samples and in different cultural contexts.

## 5. Conclusion

Our study offers evidence that retrospectively recalled memories of being raised in diversity and having substantial and meaningful intergroup experiences during childhood and adolescence (such as being raised in diversity and having intergroup friends, helping or being helped by various others, and having experiences leading to re- or de-categorization) connect to stronger IWAH in adulthood. Moreover, that evidence comes from both U.S. students and a nationally representative sample of Polish citizens. The two studies, conducted in countries with different ethno-cultural characteristics, showed that childhood/adolescent experiences, as described by the CAIE scale, significantly predicted IWAH beyond the effects of dispositional empathy, openness to experience, the value of universalism, right-wing authoritarianism, social dominance orientation, and ethnocentrism.

We believe that these results carry important implications for parents, teachers, and others who want to enlarge children’s and adolescents’ senses that all humankind is their ingroup and boost their concern for people all over the world. Generic childrearing styles, whether the parents or others are punitive or lenient, unaffectionate or not, appear to matter very little, if at all, in this development of global human identification. On the other hand, deliberately exposing children and adolescents to those of different cultures, helping them create friendships with those from distant cultures, and making them aware of common humanity among people – matters greatly. As children and adolescents develop into adulthood, these specific experiences appear to increase their IWAH, and they do so beyond children’s dispositions.

## Data availability statement

The raw data supporting the conclusions of this article will be made available by the authors, without undue reservation.

## Ethics statement

The studies involving human participants were reviewed and approved by Western Kentucky University Ethics Committee. The patients/participants provided their written informed consent to participate in this study.

## Author contributions

KH developed the study concept and design and the scale, performed the data analysis, drafted the manuscript, and made all the revisions. KH and SM performed the testing and data collection. SM provided additions and revisions to the original manuscript. All authors approved the final version of the submitted manuscript.

## Footnotes

1 Additional regression analyses for IWAH factors (bond with and concern for all humanity) are presented in Supplementary material 2. CAIE-USA predicted bond, but not concern factor.

2 A part of this sample has been used in previous studies (i.e., Sparkman and Hamer, 2020).

3 Additional regression analyses for IWAH factors (bond with and concern for all humanity) are presented in Supplementary material 2. CAIE significantly predicted both IWAH bond and IWAH concern beyond other predictors.
