# Supplementary material for: The role of early intergroup experiences for identification with all humanity in adulthood
Source: Front Psychol. 2023 Mar 15;14:1042602. doi: 10.3389/fpsyg.2023.1042602 (PMC10050495; doi:10.3389/fpsyg.2023.1042602)
Supplement: Supplementary file 1 [file Data_Sheet_1.pdf]

Supplementary Material

1 Childhood/Adolescent Intergroup Experiences (CAIE) Scale by K. Hamer

1.1. The U.S. language version (used in Study 1)

*The following statements inquire about your childhood and adolescence. For each item, indicate how well it describes you by choosing the appropriate number on the scale below. Read each item carefully before responding. Answer as honestly as you can. Thank you***.**

1. I grew up having friends from different cultural or/and ethnic background from my own.
2. My parents/caregivers taught me to be empathetic toward all people.
3. I had an "opening up" experience with people from different cultural or/and ethnic background during my childhood or adolescence.
4. During my childhood or adolescence I had an experience showing me that skin color, attractiveness, body built or other "trivial" differences between people do not really matter compared to their personality.
5. I deeply experienced suffering of someone from different cultural or/and ethnic background during my childhood or adolescence.
6. I grew up in an environment with people from different cultural or/and ethnic background present or visiting.
7. During my childhood or adolescence a person from different cultural or/and ethnic background helped me when I needed support.
8. During my childhood or adolescence a person from different cultural or/and ethnic background helped a person from my group when s/he needed support.
9. During my childhood or adolescence I did **not** like it when a person from different cultural or/and ethnic background was discriminated against.

The scale used for each item in the US:

1 - This statement does not describe me at all, 2 - This statement generally does not describe me, 3 - This statement generally describes me, 4 - This statement very much describes me

1.2. Polish language version (used in Study 2)[1]

Poniższe stwierdzenia opisują możliwe zdarzenia z przeszłości. Proszę przeczytać uważnie każde stwierdzenie, a następnie wskazać, w jakim stopniu *Pana/ią opisuje:*

1. Dorastałem/am mając przyjaciół z różnych środowisk, także odmiennych kulturowo lub etnicznie od mojego.
2. W okresie dzieciństwa lub dorastania miałem/am takie doświadczenie, które sprawiło, że stałem/łam się bardziej otwarty/a na ludzi z różnych środowisk, także odmiennych kulturowo lub etnicznie od mojego.
3. Moi rodzice lub opiekunowie uczyli mnie, by być empatycznym wobec wszystkich ludzi (czyli wczuwać się w to, co mogą myśleć lub czuć).
4. W okresie dzieciństwa lub dorastania miałem/am takie doświadczenie, które pokazało mi, że atrakcyjność fizyczna, budowa ciała, kolor skóry albo inne „powierzchowne" (zewnętrzne) różnice między ludźmi niewiele tak naprawdę znaczą w porównaniu z charakterem człowieka.
5. W okresie dzieciństwa lub dorastania głęboko przeżyłem/am sytuację, gdy dostrzegłem/am cierpienie osoby lub osób z innego kulturowo lub etnicznie środowiska niż moje.
6. Wyrastałem/am w otoczeniu, w którym byli obecni lub czasem pojawiali się ludzie z różnych środowisk, np. odmiennych kulturowo lub etnicznie.
7. W trakcie dzieciństwa lub dorastania osoba z odmiennego niż moje środowiska pomogła mi, kiedy tego potrzebowałem/am.
8. W trakcie dzieciństwa lub dorastania zdarzyło się, że osoba z odmiennego niż moje środowiska (kulturowo lub etnicznie) pomogła komuś z mojej grupy, gdy ta osoba tego potrzebowała.
9. W trakcie dzieciństwa lub dorastania **nie** podobało mi się, gdy dyskryminowano (źle traktowano) osobę z odmiennego środowiska (kulturowo lub etnicznie) niż moje.

The scale used for each item in Poland:

1 - nie opisuje mnie dobrze, 2 - raczej nie opisuje mnie dobrze, 3 - trudno powiedzieć, 4 - raczej dobrze mnie opisuje, 5 - dobrze mnie opisuje

---

[1] Since there are some differences in the order of items between English and Polish versions, please look at Supplement 4 for comparisons.

2 Supplementary regression analyses

2.1 Predictors of Identification with all humanity (Study 1)

Supplement Table 1a. Predictors of Bond with all humanity (Study 1).

	Model 1			Model 2		
Variables	β	*B (SE)*	95% CI	β	*B (SE)*	95% CI
Intercept		1.10 (0.55)	[0.01, 2.19]		0.99 (0.57)	[-0.12, 2.11]
Openness to experience	0.19**	0.21 (0.07)	[0.07, 0.35]	0.19**	0.21 (0.07)	[0.07, 0.35]
Empathy	0.03	0.04 (0.10)	[-0.15, 0.23]	0.01	0.02 (0.10)	[-0.17, 0.21]
Universalism	0.18*	0.17 (0.07)	[0.03, 0.31]	0.15*	0.15 (0.07)	[0.03, 0.29]
RWA	0.05	0.05 (0.07)	[-0.09, 0.18]	0.05	0.04 (0.07)	[-0.09, 0.18]
SDO	0.06	0.07 (0.08)	[-0.10, -0.23]	0.04	0.04 (0.08)	[-0.12, 0.20]
Ethnocentrism	-0.17*	-0.18 (0.08)	[-0.35, -0.02]	-0.14^t	-0.15 (0.08)	[-0.32, 0.01]
CAIE-USA				0.13*	0.14 (0.06)	[0.02, 0.26]
Punitive P				-0.04	-0.04 (0.07)	[-0.18, 0.10]
Unaffected P				-0.002	-0.002 (0.08)	[-0.16, 0.15]
Adjusted R^2		.12			.13	
F		*F*(6,306) =8.12***			*F*(9,303) = 6.05***	
ΔR^2		.14			.02	
ΔF					*F*(3, 303) = 1.78	

Notes. *N* =313, *** *p*<.001,** *p*<.01,* *p*<.05, $^t p$ < .1. IWAH – identification with all humanity, CAIE – Childhood/Adolescent Intergroup Experiences scale, RWA – right wing authoritarianism, SDO – social dominance orientation, Punitive P – punitive parental style, Unaffected P – unaffected parental style.

Supplement Table 1b. Predictors of Concern with all humanity (Study 1).

	Model 1			Model 2		
Variables	β	*B (SE)*	95% CI	β	*B (SE)*	95% CI
Intercept		1.59 (0.46)	[0.69, 2.49]		1.55 (0.47)	[0.62, 2.47]
Openness to experience	-0.09	-0.09 (0.06)	[-0.20, 0.02]	-0.09	-0.09 (0.06)	[-0.21, 0.03]
Empathy	0.24***	0.32 (0.08)	[0.17, 0.48]	0.23***	0.31 (0.08)	[0.16, 0.47]
Universalism	0.39***	0.34 (0.06)	[0.23, 0.46]	0.37***	0.33 (0.06)	[0.21, 0.45]
RWA	0.19**	0.17 (0.06)	[0.06, 0.28]	0.19**	0.17 (0.06)	[0.06, 0.28]
SDO	-0.08	-0.08 (0.07)	[-0.21, 0.06]	-0.09	-0.09 (0.07)	[-0.21, 0.05]
Ethnocentrism	-0.05	-0.05 (0.07)	[-0.19, 0.09]	-0.04	-0.04 (0.07)	[-0.18, 0.10]
CAIE-USA				0.06	0.06 (0.05)	[-0.04, 0.17]
Punitive P				-0.03	-0.03 (0.06)	[-0.14, 0.09]
Unaffected P				0.01	0.01 (0.07)	[-0.12, 0.13]
Adjusted R^2		.27			.27	
F		*F*(6,306) =20.52***			*F*(9,303) = 13.78***	
ΔR^2		.29			.004	
ΔF					*F*(3, 303) = 0.52	

Notes. *N* =313, *** *p*<.001,** *p*<.01,* *p*<.05, $^t p < .1$. IWAH – identification with all humanity, CAIE – Childhood/Adolescent Intergroup Experiences scale, RWA – right wing authoritarianism, SDO – social dominance orientation, Punitive P – punitive parental style, Unaffected P – unaffected parental style.

2.2 Predictors of Identification with all humanity (Study 2)

Supplement Table 2a. Predictors of Bond with all humanity (Study 2).

	Model 1			Model 2		
Variables	β	*B (SE)*	95% CI	β	*B (SE)*	95% CI
Intercept		0.89 (0.37)	[0.17, 1.61]		0.74 (0.36)	[0.03, 1.45]
Openness to experience	.19***	0.28 (0.05)	[0.18, 0.38]	.16***	0.23 (0.05)	[0.13, 0.33]
Empathy	.06	0.11 (0.07)	[-0.03, 0.24]	.04	0.08 (0.07)	[-0.05, 0.21]
Universalism	.13***	0.16 (0.05)	[0.06, 0.25]	.13***	0.16 (0.05)	[0.07, 0.25]
RWA	.003	0.004 (0.04)	[-0.07, 0.08]	-.01	-0.02 (0.04)	[-0.09, 0.06]
SDO	.05	0.12 (0.08)	[-0.04, 0.27]	$.06^{t}$	0.13 (0.08)	[-0.02, 0.28]
Ethnocentrism	-0,13***	-0.13 (0.03)	[-0.19, -0.07]	-.13***	-0.12 (0.03)	[-0.18, -0.06]
CAIE-PL				.17***	0.15(0.03)	[0.10, 0.20]
Adjusted R^2		.10			.12	
F		*F*(6, 993) = 19.12***			*F*(7, 992) = 21.07***	
ΔR^2		.10			.03	
ΔF					*F*(1, 992) = 29.48***	

Notes. *N* =1000, *** *p*<.001,** *p*<.01,* *p*<.05, $^{t}p < .1$. IWAH – identification with all humanity, CAIE – Childhood/Adolescent Intergroup Experiences scale, RWA – right wing authoritarianism, SDO – social dominance orientation, Punitive P – punitive parental style, Unaffected P – unaffected parental style.

Supplement Table 2b. Predictors of Concern with all humanity (Study 2).

	Model 1			Model 2		
Variables	β	*B (SE)*	95% CI	β	*B (SE)*	95% CI
Intercept		1.24 (0.35)	[0.55, 1.93]		1.16 (0.35)	[0.48, 1.86]
Openness to experience	.14***	0.21 (0.05)	[0.11, 0.30]	.13***	0.18 (0.05)	[0.09, 0.28]
Empathy	.12***	0.22 (0.06)	[0.09, 0.34]	.12**	0.20 (0.06)	[0.07, 0.32]
Universalism	.17***	0.20 (0.05)	[0.12, 0.29]	.17***	0.21 (0.05)	[0.12, 0.30]
RWA	<.001	-0.001 (0.04)	[-0.08, 0.07]	-.01	-0.01 (0.04)	[-0.08, 0.07]
SDO	-.01	-0.01 (0.08)	[-0.16, 0.13]	-.004	-0.01 (0.08)	[-0.16, 0.14]
Ethnocentrism	-.11***	-0.11 (0.03)	[-0.17, -0.05]	-.11***	-0.10 (0.03)	[-0.16, -0.04]
CAIE-PL				.09**	0.07 (0.03)	[0.02, 0.12]
Adjusted R^2 *F*		.13			.14	
ΔR^2		$F(6, 993) = 25.75$***			$F(7, 992) = 23.29$***	
ΔF		.14			.01	
					$F(1, 992) = 7.52$**	

Notes. *N* =1000, *** *p*<.001,** *p*<.01,* *p*<.05. IWAH – identification with all humanity, CAIE – Childhood/Adolescent Intergroup Experiences scale, RWA – right wing authoritarianism, SDO – social dominance orientation, Punitive P – punitive parental style, Unaffected P – unaffected parental style.

3 Factor analysis for the full 9-item CAIE

3.1 A. The U.S. sample (study 1)

Factor analysis with oblimin rotation was used. Although the results showed three components, we could not reasonably extract more than 1 factor because of a few reasons: 1) the rule of thumb says that each factor should have at least three variables with high loadings (see, e.g., https://www.theanalysisfactor.com/factor-analysis-5/) and this is not the case for CAIE, 2) we could not see any theoretical sense in suggested quasi factors, and 3) there is a lot of cross-loadings, even with the rotation. Thus, no clear second (or third) factor was extracted.

Supplement Table 3a. Factor analysis for the CAIE scale (Study 1).

Total Variance Explained

Component	Initial Eigenvalues			Extraction Sums of Squared Loadings			Rotation Sums of Squared Loadings[a]
	Total	% of Variance	Cumulative %	Total	% of Variance	Cumulative %	Total
1	3.564	39.595	39.595	3.564	39.595	39.595	3.278
2	1.219	13.542	53.137	1.219	13.542	53.137	1.608
3	1.007	11.189	64.326	1.007	11.189	64.326	1.882
4	.761	8.450	72.776				
5	.704	7.826	80.602				
6	.620	6.890	87.492				
7	.493	5.474	92.967				
8	.348	3.864	96.830				
9	.285	3.170	100.000				

Extraction Method: Principal Component Analysis.

a. When components are correlated, sums of squared loadings cannot be added to obtain a total variance

3.1 B. Polish sample (Study 2)

Factor analysis with oblimin rotation was used. Although, the results showed two components, we still cannot reasonably extract more than 1 factor, because of the same reasons as in the study 1.

Supplement Table 3b. Factor analysis for the CAIE scale (Study 2)

Total Variance Explained

	Initial Eigenvalues			Extraction Sums of Squared Loadings			Rotation Sums of Squared Loadings[a]
Component	Total	% of Variance	Cumulative %	Total	% of Variance	Cumulative %	Total
1	5.266	58.508	58.508	5.266	58.508	58.508	5.101
2	1.064	11.820	70.329	1.064	11.820	70.329	2.731
3	.556	6.180	76.509				
4	.535	5.943	82.451				
5	.455	5.053	87.504				
6	.371	4.122	91.626				
7	.315	3.501	95.127				
8	.268	2.973	98.100				
9	.171	1.900	100.000				

Extraction Method: Principal Component Analysis.

a. When components are correlated, sums of squared loadings cannot be added to obtain a total variance.

The tables below present very similar structure and pattern matrixes for both countries (with the first dominant factor or both; the only cases when the second factor seems more dominant are items 2 and 9). There are differences in loadings only for two items: item 3 and 4. However, the items also have substantial cross-loadings (especially in the USA).

Supplement Table 3c. Structure Matrix for the full 9-item CAIE scale (Study 1 and 2)

**Structure Matrix**	USA Component			Poland Component	
	1	2	3	1	2
1. I grew up having friends from different cultural or/and ethnic background from my own.	0.797	0.107	0.4	0.821	0.305
2. My parents/caregivers taught me to be empathetic toward all people.	0.122	0.789	-0.059	0.343	0.885
3. I had an “opening up” experience with people from different cultural or/and ethnic background during my childhood or adolescence.	0.421	0.241	0.767	0.814	0.463
4. I deeply experienced suffering of someone from different cultural or/and ethnic background during my childhood or adolescence.	0.307	-0.035	0.816	0.791	0.476
5. I grew up in an environment with people from different cultural or/and ethnic background present or visiting.	0.82	0.151	0.311	0.87	0.397
6. During my childhood or adolescence a person from different cultural or/and ethnic background helped me when I needed support.	0.858	0.192	0.312	0.873	0.356
7. During my childhood or adolescence I did not like it when a person from different cultural or/and ethnic background was discriminated against.	0.502	0.567	0.086	0.691	0.57
8. During my childhood or adolescence a person from different cultural or/and ethnic background helped a person from my group when s/he needed support.	0.808	0.233	0.256	0.868	0.345

9. During my childhood or adolescence I had an experience showing me that skin color, attractiveness, body built or other “trivial” differences between people do not really matter compared to their personality.	0.215	0.693	0.445	0.519	0.828

Extraction Method: Principal Component Analysis. Rotation Method: Oblimin with Kaiser Normalization.

Supplement Table 3d. Pattern Matrix for the full 9-item CAIE scale (Study 1 and 2)

**Pattern Matrix**[a]	USA Component			Poland Component	
	1	2	3	1	2
1. I grew up having friends from different cultural or/and ethnic background from my own.	0.767	-0.086	0.147	0.865	-0.096
2. My parents/caregivers taught me to be empathetic toward all people.	-0.022	0.806	-0.129	-0.085	0.924
3. I had an "opening up" experience with people from different cultural or/and ethnic background during my childhood or adolescence.	0.149	0.139	0.703	0.764	0.109
4. I deeply experienced suffering of someone from different cultural or/and ethnic background during my childhood or adolescence.	0.062	-0.126	0.807	0.727	0.139
5. I grew up in an environment with people from different cultural or/and ethnic background present or visiting.	-0.081	0.673	0.409	0.172	0.749
6. During my childhood or adolescence a person from different cultural or/and ethnic background helped me when I needed support.	0.817	-0.043	0.037	0.874	-0.008
7. During my childhood or adolescence I did not like it when a person from different cultural or/and ethnic background was discriminated against.	0.852	-0.009	0.023	0.902	-0.062
8. During my childhood or adolescence a person from different cultural or/and ethnic background helped a person from my group when s/he needed support.	0.426	0.478	-0.104	0.544	0.318
9. During my childhood or adolescence I had an experience showing me that skin color, attractiveness, body built or other "trivial" differences between people do not really matter compared to their personality.	0.804	0.048	-0.022	0.902	-0.073

Extraction Method: Principal Component Analysis. Rotation Method: Oblimin with Kaiser Normalization. Rotation converged in 4 iterations.

Supplement Table 3e. Component Matrix for the full 9-item CAIE scale (Study 1 and 2)

**Component Matrix**	USA			Poland	
	Component			Component	
	1	2	3	1	2
1. I grew up having friends from different cultural or/and ethnic background from my own.	0.773	-0.229	-0.105	0.787	-0.246
2. My parents/caregivers taught me to be empathetic toward all people.	0.232	0.758	-0.109	0.499	0.735
3. I had an "opening up" experience with people from different cultural or/and ethnic background during my childhood or adolescence.	0.607	0.002	0.516	0.817	-0.067
4. I deeply experienced suffering of someone from different cultural or/and ethnic background during my childhood or adolescence.	0.478	-0.24	0.629	0.8	-0.036
5. I grew up in an environment with people from different cultural or/and ethnic background present or visiting.	0.773	-0.183	-0.208	0.852	-0.179
6. During my childhood or adolescence a person from different cultural or/and ethnic background helped me when I needed support.	0.812	-0.156	-0.23	0.845	-0.227
7. During my childhood or adolescence I did not like it when a person from different cultural or/and ethnic background was discriminated against.	0.534	0.378	-0.215	0.733	0.139
8. During my childhood or adolescence a person from different cultural or/and ethnic background helped a person from my group when s/he needed support.	0.763	-0.09	-0.253	0.838	-0.235
9. During my childhood or adolescence I had an experience showing me that skin color, attractiveness, body built or other "trivial" differences between people do not really matter compared to their personality.	0.437	0.57	0.339	0.64	0.547

Extraction Method: Principal Component Analysis.

4 Childhood/Adolescent Intergroup Experiences (CAIE) Scale by K. Hamer in two versions

Supplement Table 4. Childhood/Adolescent Intergroup Experiences (CAIE) Scale by K. Hamer – analogous (parallel) items in two langage versions (Study 1 and 2)

	**English version**	**Polish version**
1.	I grew up having friends from different cultural or/and ethnic background from my own.	Dorastałem/am mając przyjaciół z różnych środowisk, także odmiennych kulturowo lub etnicznie od mojego.
2.	My parents/caregivers taught me to be empathetic toward all people.	Moi rodzice lub opiekunowie uczyli mnie, by być empatycznym wobec wszystkich ludzi (czyli wczuwać się w to, co mogą myśleć lub czuć).
3.	I had an “opening up” experience with people from different cultural or/and ethnic background during my childhood or adolescence.	W okresie dzieciństwa lub dorastania miałem/am takie doświadczenie, które sprawiło, że stałem/łam się bardziej otwarty/a na ludzi z różnych środowisk, także odmiennych kulturowo lub etnicznie od mojego.
4.	During my childhood or adolescence I had an experience showing me that skin color, attractiveness, body built or other “trivial” differences between people do not really matter compared to their personality.	W okresie dzieciństwa lub dorastania miałem/am takie doświadczenie, które pokazało mi, że atrakcyjność fizyczna, budowa ciała, kolor skóry albo inne „powierzchowne” (zewnętrzne) różnice między ludźmi niewiele tak naprawdę znaczą w porównaniu z charakterem człowieka.
5.	I deeply experienced suffering of someone from different cultural or/and ethnic background during my childhood or adolescence.	W okresie dzieciństwa lub dorastania głęboko przeżyłem/am sytuację, gdy dostrzegłem/am cierpienie osoby lub osób z innego kulturowo lub etnicznie środowiska niż moje.
6.	I grew up in an environment with people from different cultural or/and ethnic background present or visiting.	Wyrastałem/am w otoczeniu, w którym byli obecni lub czasem pojawiali się ludzie z różnych środowisk, np. odmiennych kulturowo lub etnicznie.
7.	During my childhood or adolescence a person from different cultural or/and ethnic background helped me when I needed support.	W trakcie dzieciństwa lub dorastania osoba z odmiennego niż moje środowiska pomogła mi, kiedy tego potrzebowałem/am.
8.	During my childhood or adolescence a person from different cultural or/and ethnic background helped a person from my group when s/he needed support.	W trakcie dzieciństwa lub dorastania zdarzyło się, że osoba z odmiennego niż moje środowiska (kulturowo lub etnicznie) pomogła komuś z mojej grupy, gdy ta osoba tego potrzebowała.
9.	During my childhood or adolescence I did **not** like it when a person from different cultural or/and ethnic background was discriminated against.	W trakcie dzieciństwa lub dorastania **nie** podobało mi się, gdy dyskryminowano (źle traktowano) osobę z odmiennego środowiska (kulturowo lub etnicznie) niż moje.

5 Supplementary analyses for shortened 8-item CAIE and an item about parental teaching.

Supplement Table 5. Correlations between a removed CAIE item regarding parental teaching and IWAH (Study 2).

**Variables**	**2**	**3**	**4**	**5**
1. IWAH “raw” scores	.84***	.92***	.90***	17***
2. IWAH “pure” scores	-	.79***	.75***	11***
3. IWAH bond		-	.67***	.16***
4. IWAH concern			-	.18***
5. My parents/caregivers taught me to be empathetic toward all people.				-

Notes. *N* =1000, *** *p*<.001. IWAH – identification with all humanity.

Supplement Table 6. Predictors of identification with all humanity, including shortened 8-item CAIE and a removed CAIE item regarding parental teaching (Study 2).

	Model 1			Model 2		
Variables	β	*B (SE)*	95% CI	β	*B (SE)*	95% CI
Intercept		1.05 (0.33)	[0.41, 1.69]		0.94 (0.32)	[0.30, 1.57]
Openness to experience	.19***	0.25 (0.05)	[0.16, 0.34]	.16***	0.22 (0.05)	[0.13, 0.31]
Empathy	.09*	0.15 (0.06)	[0.03, 0.27]	.08*	0.13 (0.06)	[0.01, 0.24]
Universalism	.16***	0.18 (0.04)	[0.10, 0.26]	.17***	0.19 (0.04)	[0.10, 0.27]
RWA	.003	0.003 (0.04)	[-0.07, 0.07]	-.01	-0.01 (0.04)	[-0.08, 0.06]
SDO	.03	0.06 (0.07)	[-0.08, 0.20]	.03	0.07 (0.07)	[-0.06, 0.21]
Ethnocentrism	-.14***	-0.12 (0.03)	[-0.18, -0.07]	-.13***	-0.12 (0.03)	[-0.17, -0.06]
CAIE-PL				.13***	0.11 (0.02)	[0.05, 0.16]
Parental teaching				.01	0.01 (0.02)	[-0.04, 0.05]
Adjusted R^2		.13			.15	
F		$F(6, 993) = 26.49$***			$F(7, 992) = 22.73$***	
ΔR^2		.14			.02	
ΔF					$F(2, 993) = 10.01$***	

Notes. N =1000, *** p<.001,** p<.01,* p<.05. RWA – right wing authoritarianism, SDO – social dominance orientation, CAIE – Childhood/Adolescent Intergroup Experiences scale, Parental teaching – a removed CAIE item (*My parents/caregivers taught me to be empathetic toward all people*).
